# Increasing the uptake of carbon dioxide

**DOI:** 10.7554/eLife.64380

**Published:** 2020-12-03

**Authors:** Eric Franklin, Martin Jonikas

**Affiliations:** Department of Molecular Biology, Princeton UniversityPrincetonUnited States

**Keywords:** carbon fixation, carbon-concentrating mechanism, photosynthesis, carboxysome, synthetic biology, genetic engineering, *E. coli*

## Abstract

A mechanism for concentrating carbon dioxide has for the first time been successfully transferred into a species that lacks such a process.

**Related research article** Flamholz AI, Dugan E, Blikstad C, Gleizer S, Ben-Nissan R, Amram S, Antonovsky N, Ravishankar S, Noor E, Bar-Even A, Milo R, Savage DF. 2020. Functional reconstitution of a bacterial CO_2_ concentrating mechanism in *Escherichia coli*. *eLife* **9**:e59882. doi: 10.7554/eLife.59882

Look around: how many things do you see made of wood, cloth or plastic? These items may seem wildly different, but they all contain organic carbon and, therefore, they can only exist because plants, algae and certain bacteria are constantly using photosynthesis to turn sunlight, water and atmospheric carbon dioxide (CO_2_) into most of our food, furniture and fuel (Fischer et al., 2016). However, this process has gotten more difficult over time. Modern CO_2_ levels are less than 1% of what they were when photosynthetic organisms first evolved, making the work of Rubisco, the enzyme that converts CO_2_ into organic molecules, more difficult. In turn, the slow rate of CO_2_ uptake limits the growth of many plants, including crops such as rice and wheat (Long et al., 2006).

Some organisms, however, have evolved ways to concentrate CO_2_ around Rubisco, allowing the enzyme to run faster (Hennacy and Jonikas, 2020). Introducing such carbon-concentrating mechanisms into crops could increase yields by 60% while reducing water and fertilizer requirements (McGrath and Long, 2014). The best understood carbon-concentrating mechanism is the one found in bacteria, which is based on a protein structure called the ‘carboxysome’ that contains Rubisco and other carbon fixation-related enzymes. These species actively import carbon in the form of bicarbonate (HCO_3_^–^), which diffuses into the carboxysome and is converted to CO_2_. The resulting high CO_2_ concentration achieved within the carboxysome maximizes the activity of Rubisco and therefore increases overall CO_2_ uptake (Figure 1A).

**Figure 1.**
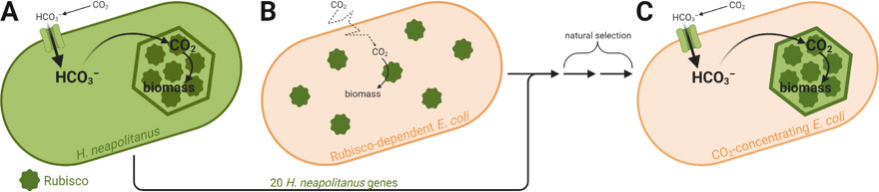
Engineering a carbon-concentrating mechanism into *E. coli*. (**A**) *Halothiobacillus neapolitanus* has a carbon-concentrating mechanism that relies on structures called carboxysomes. The cell imports CO_2_ as bicarbonate (HCO_3_^–^), which diffuses into the carboxysome (green hexagon) and is converted into concentrated CO_2_. The elevated levels of CO_2_ in the carboxysome allow the enzyme Rubisco (dark green) to convert it to biomass more efficiently. (**B**) Flamholz et al. engineered a strain of *E. coli* to be dependent on Rubisco activity for its growth. Rubisco runs slowly in this strain as it can only use CO_2_ which diffuses (dotted line) into the cell from the atmosphere. (**C**) However, adding just 20 genes from *H. neapolitanus* and selecting for cells that can grow in low levels of CO_2_ led to an *E. coli* strain with a reconstituted *H. neapolitanus* carbon-concentrating mechanism based on carboxysomes, which allows Rubisco to run much faster.

Figure created with BioRender.com.

Previous work managed to assemble carboxysome-like structures in the non-photosynthetic model bacterium *Escherichia coli* (Bonacci et al., 2012). However, these cells required high levels of CO_2_ for growth, indicating that additional components were required to concentrate CO_2_. Now, in eLife, David Savage, Ron Milo and colleagues – including Avi Flamholz as first author – report how they have engineered a functional carbon-concentrating mechanism into an organism that lacks one (Flamholz et al., 2020).

The team, which is based at the University of California, Berkeley, the Weizmann Institute of Science and the Max Planck Institute of Molecular Plant Physiology, chose the bacterium *Halothiobacillus neapolitanus* as the genetic donor for their experiment. Carboxysomes in this species are simple and well-studied: in particular, Savage and co-workers had previously identified 20 candidate genes likely needed for these structures to work properly (Desmarais et al., 2019).

As their recipient species, Flamholz et al. chose *E. coli*, which they genetically modified to rely on Rubisco’s activity for growth (Figure 1B). Without a carbon-concentrating mechanism, this strain could not grow in ambient air – it required supplementation with CO_2_ levels about 100 times higher than those found in the atmosphere. Hoping to reconstitute a functional carbon-concentrating mechanism, the team transferred the 20 candidate genes from *H. neapolitanus* to their *E. coli* strain. Unsurprisingly, the strain was still unable to grow in ambient CO_2 _at first, as simply adding genes is often not enough to engineer a complex pathway into a new organism (Antonovsky et al., 2016).

However, Flamholz et al. were able to leverage an important feature of their genetically engineered *E. coli* strain – its growth rate is proportional to Rubisco’s activity. This allowed the team to use a natural selection experiment to spot mutations that make the carbon-concentrating mechanism work, and therefore increase Rubisco activity. The experiment revealed a mutant that could grow at ambient CO_2_ levels, apparently by adjusting the expression levels of the proteins taking part in the carbon-concentrating process.

This result suggested that a carbon-concentrating mechanism based on *H. neapolitanus* carboxysomes had successfully been reconstituted in their *E. coli* strain (Figure 1C). To further support this conclusion, electron microscopy was used to observe the carboxysome-like structures within the engineered *E. coli* strain. To make sure these structures were functional, they individually knocked out several genes known to be essential for carboxysome function in the native host. These mutations had the same effect in *E. coli* as in *H. neapolitanus* – the cells no longer grew at ambient CO_2_ levels – confirming that the carboxysome was working the same way in the engineered strain as in the native host.

These results from Flamholz et al. indicate that a carboxysome-based carbon-concentrating mechanism can be transferred and function in another organism, providing a blueprint that paves the way toward engineering plants with increased CO_2_ uptake and thus greater yields.
